# Upright epiglottis prevents aspiration in patients with nasopharyngeal carcinoma post-chemoradiation

**DOI:** 10.1371/journal.pone.0261110

**Published:** 2021-12-09

**Authors:** Susyana Tamin, Marlinda Adham, Arfan Noer, Nana Supriana, Saptawati Bardosono

**Affiliations:** 1 Department of Otorhinolaryngology-Head and Neck Surgery, Faculty of Medicine, Universitas Indonesia – Cipto Mangunkusumo Hospital, Jakarta, Indonesia; 2 Department of Radio Oncology, Faculty of Medicine, Universitas Indonesia – Cipto Mangunkusumo Hospital, Jakarta, Indonesia; 3 Department of Nutrition Science, Faculty of Medicine, Universitas Indonesia – Cipto Mangunkusumo Hospital, Jakarta, Indonesia; Sapienza University of Rome, ITALY

## Abstract

NPC is the most widely found malignant tumor in the head and neck region in Indonesia. Chemoradiation therapy for NPC can induce swallowing disorders (dysphagia) that adversely affects a patients quality of life. This study aimed to assess the swallowing process by flexible endoscopic evaluation of swallowing in patients with nasopharyngeal carcinoma after chemoradiation. Thirty-nine patients with NPC who had chemoradiation therapy more than one month previously underwent flexible endoscopic evaluation of swallowing and were assessed for oral transport time, sensation, standing-secretion, pre-swallowing leakage, residue, penetration, aspiration, and silent aspiration. The most common structural abnormalities were an upright and swollen epiglottis (89.4%), poor oral hygiene, and velopharyngeal closure defects (56.4%). This examination also revealed a mild degree of standing secretion (38.5%) and aspiration (10.3%). No penetration was observed in 64.1% of the patients, and no silent aspiration was observed in any of the patients. A severe degree of residue (45.7%) was observed when administering oatmeal, while the residue was mild to moderate when administering gastric rice, crackers, and milk. The residue changed to a mild degree (32.3%–51.4%) in all food administrations after the watering maneuver. The highest penetration was noted after oatmeal administration (42.8%), and the highest aspiration was found after milk administration (8.6%). Standing secretion in almost all patients was caused by hyposensitivity of the hypopharynx. Persistent residue and hyposensitivity of the hypopharynx led to aspiration. The low percentage of aspiration and silent aspiration might have been caused by the upright and swollen epiglottis that prevented aspiration. Poor oral hygiene and a dry mouth led to prolonged oral transport. Therefore, most patients had hypopharyngeal abnormalities in the form of a swollen and upright epiglottis. Secretion and food residue were also detected. Drinking helps to expedite the swallowing process by facilitating oral phase transport and reducing residues.

## Introduction

Nasopharyngeal carcinoma (NPC) is one of the most common head and neck malignancies [1]. NPC is the most widely found malignant tumor in the head and neck region in Indonesia [2]. The treatment of NPC is complex because of its proximity to critical structures. Surgery is performed only for histological biopsy and treatment of recurrent or persistent diseases. Radiotherapy remains the main treatment for NPC because of its high radiosensitivity. Chemotherapy also plays a role in the management of NPC as an induction, concurrent, or adjuvant therapy [3]. The combination of radiation and chemotherapy in NPC may affect structures adjacent to the tumor, thus influencing its function. An early and long-term complication of NPC treatment is dysphagia or swallowing dysfunction [4]. Treatment for both demolitive and organ-conserving head and neck cancers can induce long-term swallowing disorders (dysphagia) that adversely affects a patients quality of life [5].

Based on these causes, dysphagia can be divided into mechanical obstruction and neuromuscular dysfunction. The mechanical obstruction causes of dysphagia include intrinsic structural lesions (esophageal cancer, foreign bodies, lower esophageal rings, and esophageal webs) and extrinsic structural lesions (vascular compression, lymphadenopathy, substernal thyroid, and mediastinal masses) [6]. Malnutrition develops in 79% of patients with esophageal cancer, representing the most nutritionally compromised group of patients with cancer. Dysphagia and body weight loss of ≥10% are already present at the time of diagnosis, and treatments for esophageal cancer also contribute significantly to the development of malnutrition. Nutritional support in patients with esophageal cancer is performed using a parallel therapeutic route [7]. Neuromuscular dysfunction that causes dysphagia includes diseases of the central nervous system (cerebrovascular accident, Parkinson’s disease, and brain stem tumors), degenerative disease, peripheral nervous system, and skeletal muscle diseases [6]. The pathophysiology of radiation-induced dysphagia includes a broad spectrum of structural, mechanical, and neurological deficits. Understanding the biomolecular effects of radiation on soft and nerve tissue injuries, wound healing, and underlying tissue morphological responses will improve the options available for dysphagia treatment [5].

A careful history and examination, imaging, videofluoroscopy (VF), and flexible endoscopic evaluation of swallowing (FEES) are needed to diagnose and treat dysphagia [8]. The evaluation of swallowing with baryte meal using VF is a quick and easy method, which allows the evaluation of pharyngoesophageal motility, providing useful clinical information necessary for the diagnostic setting [9]. However, VF has some disadvantages, including cost, radiation exposure, limited positioning, and transport to radiology [10]. Meanwhile, FEES is a more tolerable and comfortable instrumental assessment for patients and is sensitive for evaluating residue, aspiration, laryngeal penetration, and premature spillage. Moreover, FEES can evaluate vocal cord and pharyngeal dysfunction and changes in intraluminal and mucosal structures [11]. Other advantages of FEES over VF include the use of real food during testing [12].

In Indonesia, no data revealing the characteristics of dysphagia and the swallowing process in patients with NPC post-chemoradiation as evaluated by FEES, exist. Hence, this study aimed to assess the swallowing process in patients with NPC who underwent chemoradiation using FEES.

## Materials and methods

The study was conducted at the Integrated Dysphagia Clinic of Endoscopic Broncho-Esophagology and Oncology Head and Neck Division of the Department of Faculty of Medicine, Universitas Indonesia (FMUI)/Cipto Mangunkusumo Hospital (RSCM), Jakarta, Indonesia. This descriptive cross-sectional study assessed the swallowing process of dysphagia in patients with NPC after chemoradiation. A consecutive sampling technique was used in this study. This study was approved by the Committee of Medical Research Ethics of the Faculty of Medicine, Universitas Indonesia, with regard to the protection of human rights and welfare in medical research. The participants provided written and verbal informed consent before study participation.

**Fig 1:**
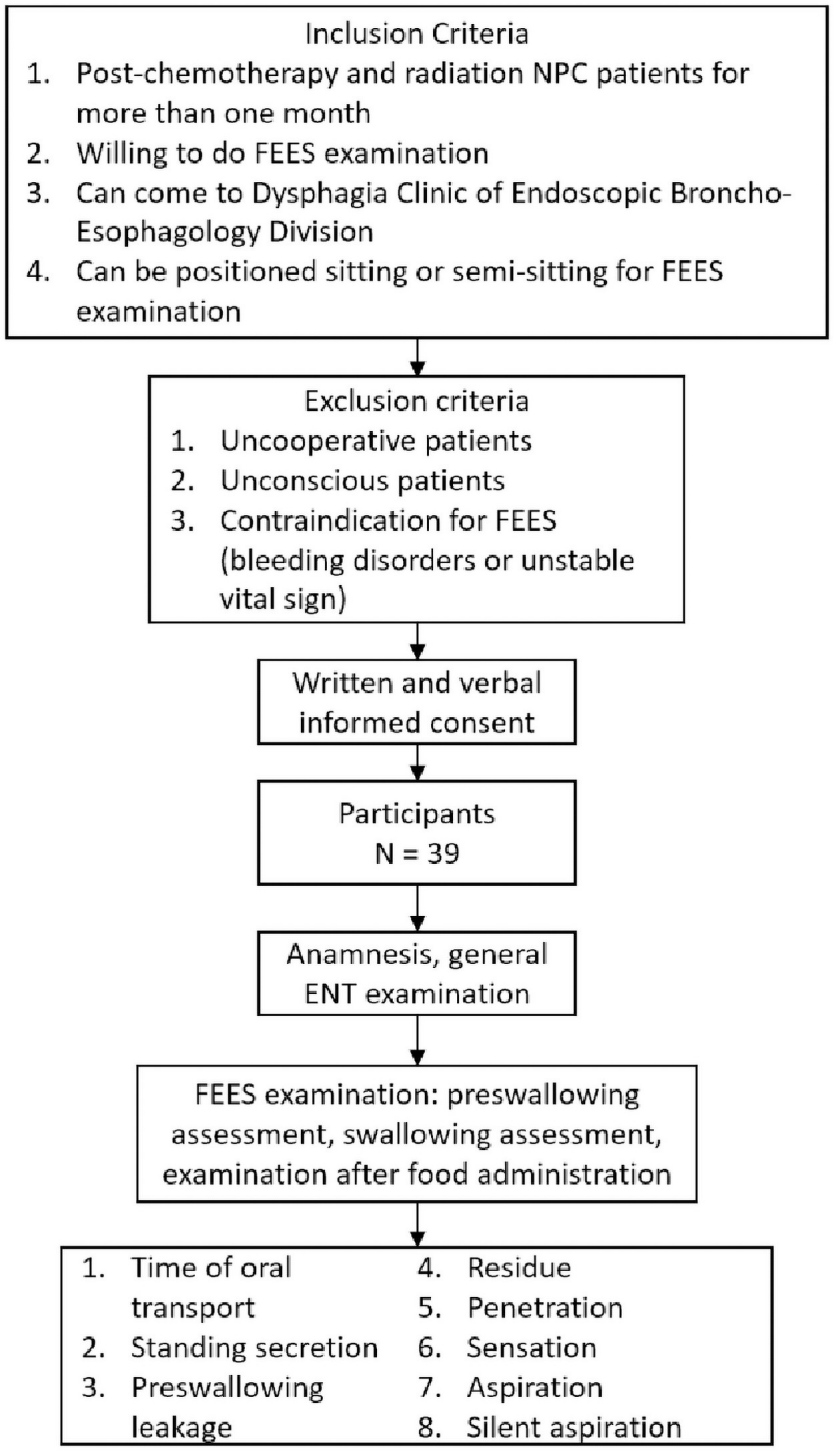
Study flowchart.

The study included patients of all ages with NPC who underwent chemotherapy and radiation therapy more than one month previously and came to the Integrated Dysphagia Clinic of the ENT Department of FMUI/RSCM, Oncology Clinic ENT Department of FMUI/RSCM, and Radiotherapy Clinic of FMUI/RSCM with or without complaints of dysphagia who met the inclusion criteria. The inclusion criteria included post-chemotherapy and radiation dose more than one month previously, willingness to undergo FEES examination and sign the consent letter, can come to the Dysphagia Clinic of Endoscopic Broncho-Esophagology Division, and can be positioned sitting or semi-sitting for FEES examination. The exclusion criteria included uncooperative, unconscious, or patients with contraindications for FEES examination, such as bleeding disorders or unstable vital signs. Altogether, 39 patients with NPC met the inclusion criteria (Fig 1). Patients underwent complete anamnesis, general ENT examination, and FEES examination to assess the time of oral transport, standing secretion, pre-swallowing leakage, residue, penetration, sensation, aspiration, and silent aspiration. The pre-swallowing assessment was performed to evaluate the structure involved in the swallowing process, the swallowing assessment was performed to evaluate the swallowing process before food administration, and the FEES examination was conducted after administration of five kinds of food with different consistencies, namely puree, gastric rice, oatmeal, crackers, and milk. The FEES examination was performed by an ENT specialist and staff of the Endoscopic Broncho-Esophagology Division FMUI using fiberoptic nasopharyngolaryngoscopy Olympus visera ENF type V. The stadium of the NPC was classified based on the Union International Cancer Committee 2002.

## Results

Of the 39 participants, as shown in Table 1, most of them were male. The distribution of age-based samples was divided into four age groups, in which the most prevalent samples were in the age group >40 years (25 patients or 64.1%) with the youngest age span of 16 years and the oldest age of 64 years. This study found that the number of patients with advanced tumor stage (III–IV) was the highest (84.6%). The most common oral phase complaints were dry mouth in 36 patients (92.3%), whereas in the pharynx phase the proportion of complaints was 28.2%. Furthermore, the type of radiotherapy used in all patients was non-intensity-modulated radiotherapy (non-IMRT).

**Table 1:** Baseline demographic and clinical characteristics of the patients.

Characteristics		Amount (%)
**Sex**	Male	24 (61.5)
	Female	15 (38.5)
**Age groups (years)**	<40	14 (35.9)
	40–49	13 (33.3)
	50–59	9 (23.1)
	≥60	3 (7.7)
**Tumor stage**	I–II (early)	6 (15.4)
	III–IV (advanced)	33 (84.6)
**Complaints**	**Oral phase**	
	Dry mouth	36 (92.3)
	Swallowing pain	10 (25.6)
	Sense of suffocated	8 (20.5)
	Chewing disorder	12 (30.8)
	Tooth decay	10 (25.6)
	Loss of taste	17 (43.8)
	**Pharynx phase**	
	Cough/choking	11 (28.2)
	Globus sensation	11 (28.2)
	Prolonged of meal	11 (28.2)

Initial assessments with FEES were performed to evaluate the anatomical structures involved in the swallowing process. The complete results of these examinations are shown in Table 2. The most common abnormalities were swollen and upright epiglottis in 35 patients (89.4%), followed by poor oral hygiene and inadequate velopharyngeal closure during swallowing in 22 patients (56.4%).

**Table 2:** The results of the initial assessment with FEES (n = 39).

Characteristics		Amount (%)
Poor oral hygiene		22 (56.4)
Tongue muscle weakness		1 (25.6)
Tongue fasciculation		9 (23.1)
Buccal muscle weakness		2 (5.1)
Asymmetric movement of soft palate		12 (30.8)
Inadequate closure of velopharyngeal during swallowing		22 (56.4)
Swollen and upright epiglottis		35 (89.4)
Laryngeal edema	Mild	7 (17.9)
	Moderate	10 (25.6)
	Severe	18 (46.2)
Poor cough reflex		12 (30.8)
Absence of voluntary swallow		17 (43.6)

Table 3 shows the FEES findings prior to the food administration. The highest degree of standing secretion was mild (38.5%). Our study did not find penetration in 64.1% of the patients, as aspiration events were only detected in four patients (10.3%). No silent aspiration was observed on the FEES examination.

**Table 3:** Swallowing assessment with FEES.

Characteristics		Amount (%)
Standing secretion	Normal	3 (7.7)
	Mild	15 (38.5)
	Moderate	9 (23.1)
	Severe	12 (30.8)
Penetration	0	25 (64.1)
	1	2 (5.1)
	2	10 (25.6)
	3	1 (2.6)
	4	1 (2.6)
Aspiration		4 (10.3)
Silent aspiration		–

Table 4 shows the results of the FEES examination on the administration of various kinds of food. In puree, the highest residue was found at a moderate degree in 43.2% of the patients. After the watering maneuver, they turned to a mild degree with the highest number (37.8%). Of the total number of patients, 59% did not exhibit penetration, and only 8.1% indicated aspiration. Only 1 patient (2.7%) had silent aspiration.

**Table 4:** The results of swallowing assessment by FEES after foods administration.

Characteristics		Puree (n = 37)	Gastric rice (n = 36)	Oatmeal (n = 35)	Crackers (n = 31)	Milk (n = 35)
**Residue**	Normal	2 (5.4)	–	–	1 (3.2)	3 (5.7)
	Mild	5 (13.5)	8 (22.2)	10 (28.6)	5 (16.1)	14 (40.0)
	Moderate	16 (43.2)	18 (50.0)	9 (25.7)	17 (54.8)	9 (25.7)
	Severe	14 (37.8)	10 (27.8)	16 (45.7)	8 (25.8)	10 (28.6)
**Residue post-watering maneuver**	Normal	7 (18.9)	8 (22.2)	8 (22.9)	10 (32.3)	10 (28.6)
	Mild	14 (37.8)	16 (44.4)	17 (48.6)	10 (32.3)	18 (51.4)
	Moderate	10 (27.0)	9 (25.0)	7 (20)	7 (22.6)	5 (14.3)
	Severe	6 (16.2)	3 (8.3)	3 (8.6)	4 (17.9)	2 (5.7)
**Penetration**	0	22 (59.5)	22 (61.1)	20 (57.1)	19 (61.3)	24 (68.6)
	1	2 (5.4)	2 (5.6)	6 (17.1)	3 (9.7)	3 (8.6)
	2	12 (32.4)	11 (30.6)	9 (25.7)	9 (29.0)	8 (22.9)
	3	1 (2.7)	1 (2.8)	–	–	–
	4	–	–	–	–	–
**Aspiration**		3 (8.1)	3 (8.3)	2 (5.7)	1 (3.2)	3 (8.6)
**Silent aspiration**		1 (2.7)	1 (2.8)	1 (2.9)	1 (3.2)	1 (2.9)

In the administration of gastric rice, a moderate degree of residue was observed in 50% of the patients, which changed to a mild degree after the watering maneuver in 44.4%. No penetration was observed in 61.1% of the patients. Aspiration was recorded in 8.3% of the patients, as silent aspiration was found only in one patient or 2.8%.

In the administration of oatmeal, a severe degree of residue was found in the highest number of 45.7% of the patients and after the watering maneuver, the degree of residue was observed to be mild in the highest number of 48.6%. Aspiration occurred in two patients (5.7%), and silent aspiration occurred in one patient (2.9%).

In the administration of crackers, our study found a moderate degree of residue in 54.8%, and after the watering maneuver, the residue changed to a mild degree and normal in the highest number of 32.3%. Silent aspiration occurred in one patient (3.2%).

In the administration of milk, the mild residue was found in the highest amount (40.0%), and after the watering maneuver, the residue was reduced to a mild degree in the highest number (51.4%). Aspiration occurred in three patients (8.6%), and silent aspiration occurred in one patient (2.9%).

Table 5 describes the distribution of the FEES examination results based on tumor stage, type of chemotherapy, and radiation dose. In the early stages, a moderate degree of standing secretion was observed in 66.7% of the patients, and no severe standing secretion was noted. However, the incidence of standing secretion at an advanced stage was higher in severe cases (27.3%). As shown in Table 5, no increase in the incidence rate of penetration based on tumor stage, type of chemotherapy, and radiation dose was observed. Hence, the incidence of aspiration is very low (<50%) based on tumor stage, type of chemotherapy, and radiation dose.

**Table 5:** FEES finding characteristics based on tumor stage, chemotherapy type, and radiation dose.

Variable		Degree of standing secretion				Degree of penetration					Aspiration	
		Normal	Mild	Moderate	Severe	0	1	2	3	4	Yes	No
		n (%)	n (%)	n (%)	n (%)	n (%)	n (%)	n (%)	n (%)	n (%)	n (%)	n (%)
**Stadium**	Early (n = 6)	0	2 (33.3)	4 (66.7)	0	1 (16.7)	0	4 (66.7)	1 (16.7)	0	1 (16.7)	5 (83.3)
	Advance (n = 33)	3 (9.1)	13 (39.4)	8 (24.2)	9 (27.3)	24 (72.7)	2 (6.1)	6 (18.2)	0	1 (3)	3 (9.1)	30 (90.9)
**Chemotherapy types**	Neoadj/adj chem-rad (n = 15)	2 (13.3)	4 (26.7)	6 (40)	3 (20)	12 (80)	1 (6.7)	2 (13.3)	0	0	1 (16.7)	14 (93.3)
	Chemoradiation (n = 22)	1 (4.5)	9 (40.9)	6 (27.3)	6 (27.3)	11 (50)	1 (4.5)	8 (36.4)	1 (4.5)	1 (4.5)	3 (13.6)	19 (86.4)
	Chemotherapy (n = 2)	0	2 (100)	0	0	2 (100)	0	0	0	0	0	2 (100)
**Radiation dose**	< 70 (n = 19)	3 (15.8)	6 (31.6)	6 (31.6)	4 (21.1)	12 (63.2)	2 (10.5)	5 (26.3)	0	0	2 (10.5)	17 (89.5)
	>70 (n = 20)	0	9 (45)	6 (30)	5 (25)	13 (65)	0	5 (25)	1 (5)	1 (5)	2 (10)	18 (90)

Additionally, a prolonged oral transport time and laryngeal hyposensitivity were observed in all the patients. Our study did not identify any pre-swallowing leakage.

## Discussion

Of the 39 patients, the male sex was the most predominant (61.5%) (Table 1). A previous study has reported a male predominance in NPC incidence with a male and female incidence rate ratio of 2.36 [13]. Another survey in China, which has a high prevalence for NPC, has reported that the ratio of male to female patients was 2–5:1 [14]. The male predominance in NPC incidence may be influenced by lifestyle and environmental risk factors such as alcohol consumption and smoking, which men more often do [15, 16].

Herein, we found that most patients with NPC who come to the Department of ENT RSCM are in the advanced stage (III–IV). NPC is difficult to detect early because of its anatomic isolation and clinical silence [17]. Since most patients in Indonesia came in the late stage, the early detection system may not be adequate [18]. Abdullah has mentioned that the reasons why patients diagnosed with such a late stage of NPC include the following: a delay in seeking medical advice, the difficulty of a clinical examination of the nasopharynx, even for experienced clinicians, and the silent submucosal lesion with a normal appearance during examination of the nasopharynx. Late detection of NPC results in challenging management and poor prognosis [19].

In this study, the initial assessment through the FEES examination revealed the presence of a swollen and upright epiglottis in 35 patients (89.4%), as shown in Table 2. These results are conflicting with a previous research conducted abroad, wherein the most common abnormal anatomy occurred in patients with NPC were tongue atrophy and vocal cord paralysis [20], while in this study, abnormalities in the tongue were tongue weakness and fasciculation in 48.7% of the patients.

In the examination of swallowing function using the FEES, our study found that standing secretion occurred in almost all patients before food administration (92.3%). Standing secretion is the first FEES parameter that can be assessed in the hypopharynx. This occurs because of hypopharyngeal hyposensitivity [21]. The sensory receptor is important for efficiently transporting the bolus into the pharynx [22].

After food administration, most residues arose in the vallecula. The residue would be reduced in amount after the watering maneuver. This result is consistent with our study, where food residue was found in the vallecula in all the patients (100%) and piriformis sinuses (60%). Another study also found food stasis in the pharynx (60% in pyriform fossae and 100% in valleculae) after 12 months of radiotherapy. All participants (100%) also needed a drink to wash food down compared to before radiotherapy (20%). Persistent residue in the larynx may lead to an overflow of aspiration and subsequent chest complications [23].

Herein, the incidence of penetration and aspiration was not >50%, the highest penetration rate in food oatmeal was 42.8%, and it was only 8.6% for aspiration. This result is very different from that of Wu’s study, which found penetration and aspiration in 93.5% of the patients. Silent aspiration in this study occurred in only 3.2% of the patients, while Wu found silent aspiration in up to 41.9% of the patients [24]. Another study by Ng et al. has reported that silent aspiration occurred in 65.9% of patients. The frequency of silent aspiration increased as viscosity decreased by 5.9% for soft diet, 11.8% for pureed diet, 35.3% for thick fluids, and 64.7% for thin fluids [25]. Different results are possible because anatomical abnormalities in Wu’s study are in the form of tongue atrophy and vocal cord paralysis, which can lead to aspiration [24]. Here, the most anatomical abnormality was a swollen and upright epiglottis. A study by Seo et al., which investigated swallowing kinematics related to penetration-aspiration post-stroke patients with dysphagia, has indicated that the maximal tilt angle of the epiglottis was lower in patients with aspiration than that in patients without penetration or aspiration [26].

Prolonged oral transport times occurred in all patients, presumably due to poor oral hygiene (56.4%) and dry mouth (92.3%). Ku et al. in their study also observed a prolonged food transport time in patients after radiation dose. The disorder is caused by the effects of radiation on soft tissues in the mouth and pharynx, as well as decreased saliva that can help transport the bolus to the pharynx [23]. Furthermore, in this study, all patients used non-IMRT as radiotherapy, with most of the usual doses being >70 Gy. Nutting et al. have reported a significant reduction in xerostomia in patients treated with IMRT compared with conventional radiotherapy [27]. A significant reduction in GD can be found in patients with radiation levels of >52 Gy [28]. Pauloski et al. have reported that patients treated with IMRT demonstrated shorter pharyngeal and oral transport times than those treated with conventional radiotherapy [29].

Herein, the absence of pre-swallowing leakage may be due to the apparent weakness of the tongue and asymmetric movement of the soft palate (25.6% and 30.8%, respectively). Bolus leakage into the oropharynx before the swallow was prevented by contact with the tongue and soft palate, which sealed the posterior oral cavity. Weak contraction of the soft palate and tongue may lead to premature leakage of the bolus, especially liquids [30].

The senile evolution of post-surgical laryngeal function or post-radiochemotherapy treatment is different from the paraphysiological reduction in swallowing in elderly patients with presbyophagy. Parameters such as the dose of therapy administered or the amount of resection and the degree of possible impairment of swallowing and phonatory parameters have been shown to be significantly correlated [31, 32].

Cancer treatments such as curative radiotherapy and chemoradiotherapy for head and neck cancers can cause long-term swallowing disorders such as dysphagia, which negatively affect quality of life by acting on different structural, mechanical, and neurological deficits. The complexity of the pathophysiology of radiation-induced injury and the areas need further exploration to clarify concepts and lay the foundations for new therapeutic approaches [5].

Various options such as new radiotherapy techniques, compliance with maximum dose guidelines for organs involved in swallowing, functional rehabilitation, and evaluation of swallowing are available to reduce the risk of dysphagia after chemoradiotherapy and thus prevent aspiration [33].

Oropharyngeal surgery, which is highly invasive, has always presented with a significant complication of oropharyngeal dysphagia, which can significantly affect a patient. Hence, organ preservation with definitive chemoradiotherapy is often preferred. The introduction of robotic surgery has allowed the use of minimally invasive approaches, reducing hospitalization times, postoperative bleeding, and dysphagia complications [34].

## Conclusion

Changes in the hypopharyngeal structure were observed in the form of a swollen and upright epiglottis in 89.4% of the patients with NPC after chemotherapy and radiation dose. Dysphagia findings based on FEES did not demonstrate pre-swallowing leakage, low prevalence of penetration, and an insignificant number of aspirations and silent aspirations. In contrast, the prevalence of standing secretion, food residues, and prolonged oral and pharyngeal phase transport times was very high. Drinking helps to expedite the swallowing process, especially facilitating oral phase transport and reducing residues.
